# Particle Backtracking Improves Breeding Subpopulation Discrimination and Natal-Source Identification in Mixed Populations

**DOI:** 10.1371/journal.pone.0120752

**Published:** 2015-03-23

**Authors:** Michael E. Fraker, Eric J. Anderson, Reed M. Brodnik, Lucia Carreon-Martinez, Kristen M. DeVanna, Brian J. Fryer, Daniel D. Heath, Julie M. Reichert, Stuart A. Ludsin

**Affiliations:** 1 Aquatic Ecology Laboratory, Department of Evolution, Ecology and Organismal Biology, The Ohio State University, Columbus, Ohio, United States of America; 2 NOAA-GLERL, Ann Arbor, Michigan, United States of America; 3 Department of Biology, University of Texas at Brownsville, Brownsville, Texas, United States of America; 4 GLIER, University of Windsor, Windsor, ON, N9B 3P4, Canada; National Taiwan University, TAIWAN

## Abstract

We provide a novel method to improve the use of natural tagging approaches for subpopulation discrimination and source-origin identification in aquatic and terrestrial animals with a passive dispersive phase. Our method integrates observed site-referenced biological information on individuals in mixed populations with a particle-tracking model to retrace likely dispersal histories prior to capture (i.e., particle backtracking). To illustrate and test our approach, we focus on western Lake Erie’s yellow perch (*Perca flavescens*) population during 2006–2007, using microsatellite DNA and otolith microchemistry from larvae and juveniles as natural tags. Particle backtracking showed that not all larvae collected near a presumed hatching location may have originated there, owing to passive drift during the larval stage that was influenced by strong river- and wind-driven water circulation. Re-assigning larvae to their most probable hatching site (based on probabilistic dispersal trajectories from the particle backtracking model) improved the use of genetics and otolith microchemistry to discriminate among local breeding subpopulations. This enhancement, in turn, altered (and likely improved) the estimated contributions of each breeding subpopulation to the mixed population of juvenile recruits. Our findings indicate that particle backtracking can complement existing tools used to identify the origin of individuals in mixed populations, especially in flow-dominated systems.

## Introduction

Identifying dispersal patterns and the degree of connectivity among local breeding subpopulations, as well as the natal source(s) of dispersed individuals in a mixed population, can improve our ability to understand the structure and dynamics of aquatic and terrestrial populations [1–2]. In turn, such understanding can have applied benefits. Most obvious has been the enhancement of species conservation strategies wherein knowledge of dispersal pathways and subpopulation connectivity has guided the placement of dispersal corridors and the design of reserve networks [3–4]. Knowledge of the compositional makeup of a mixed population also can improve the effectiveness of resource management [5–6] by allowing for the development of harvest allocation strategies that protect important breeding subpopulations [5] and by identifying underperforming subpopulations that might be in need of rehabilitation [7]. Likewise, the identification of key or unexpected sources of individuals to a mixed population can help with the control or mitigation of native pests, invasive species, or disease [8–11].

To enhance our ability to address questions related to dispersal, population connectivity, and population structure and dynamics, much effort has been spent developing tools that can discriminate among local breeding subpopulations [12–13]. This need is paramount because mixing of local breeding subpopulations is common during one or more life stages for many species, including aquatic and terrestrial plants [14], invertebrate animals [15], and vertebrates [16] (but see also [3]). While artificial tags continue to offer a viable means to discriminate among breeding subpopulations in many systems, technological advances made during the past two decades have increased the use of natural tags [13].

To be a reliable natural marker, it needs to be consistently found in individuals originating from a particular location, must vary among breeding sources, and must remain unchanged during ontogeny. Otherwise, the link to the natal site would be lost [12–13]. Several types of natural tags have been explored as breeding subpopulation markers, including body structures (typically “hard parts”) that can record chemical or isotopic signatures from the environment in which an individual resides [13,17–20] and various genetic markers [13,19–20].

While the approaches described above have been used in solo and combination with varying levels of success, each is potentially limited by the fundamental assumption that the individuals used to develop functions that can discriminate among local breeding subpopulations originated at their collection site. For some organisms, this assumption may be quite realistic (e.g., organisms that breed in an upstream river that is far removed from the downstream mixing zone). For others, the possibility exists that an individual collected in one breeding location may not have originated there, but instead passively or actively dispersed to its collection location from a different source (breeding) location. Such a possibility seems especially plausible in situations in which a local breeding population reproduces in or near a mixing zone, or in cases where the early life stages of a species are highly susceptible to passive dispersal by physical forces such as wind or water circulation (e.g., some fishes, aquatic and terrestrial invertebrate animals, and plants). In these systems, failure to account for pre-collection dispersal could lead to sub-par discrimination abilities that might suggest a technique is not useful (when it actually might be). Or worse, the imprecise or inaccurate discrimination functions developed might provide misleading ecological insights that then drive the development of inappropriate management or conservation strategies.

Given these concerns, we suggest complementing natural tagging approaches with information on pre-collection dispersal, which can be obtained from particle backtracking (hindcasting) approaches wherein hydrodynamic or atmospheric models are used to recreate probabilistic dispersal histories prior to collection [21]. Combining particle backtracking with natural tagging seems especially useful when considering that hydrodynamic and atmospheric models are becoming increasingly accurate and have been calibrated and validated for many ecosystems [22–24]. By integrating these approaches, the source-origin of individuals that are used to develop the breeding subpopulation discrimination and classification functions can be confirmed or revised as needed, thus eliminating the assumption of natural tagging approaches that the collection site of young individuals is equivalent to the natal (source-origin) site [18, 25–27].

Particle backtracking involves using water or wind circulation computer simulation models to retrace the most likely path a dispersed individual has taken [11, 21, 28]. Backtracking is currently most useful when individuals can be considered to be passively dispersed particles (e.g., small propagules such as eggs, seeds, or small larvae), as individual behavior can strongly influence dispersal and often is difficult to accurately include in a model [29–30]. Theoretically, when dispersal histories are combined with information from a natural marker and other relevant biological information (e.g., particle age), the reliability of initial assignment should improve, potentially leading to an increased ability to discriminate among breeding subpopulations and determine the natal origin of individuals in a mixed population.

Here, we provide an example of how particle backtracking simulations can improve the use of two commonly used natural markers—otolith micro-elemental composition and microsatellite DNA [31–32]—to discriminate among yellow perch (*Perca flavescens*) breeding subpopulations and determine their relative contribution of recruits to the age-0 juvenile stage of in the western basin of Lake Erie (USA-Canada), a system with strong river- and wind-driven water currents [33]. Because our primary intention is to illustrate the potential of backtracking to improve discrimination and assignment capabilities rather than to precisely delineate yellow perch subpopulation structure, we used intentionally broad subpopulation assignments (i.e., north shore and south shore). Towards this end, we first use larval yellow perch collection location to develop functions to discriminate between our two geographically distinct breeding subpopulations [27]. We then use these functions to assign (unknown-origin) juveniles from the mixed, open-lake population to one of the two breeding (hatching) locations. Finally, we show how combining probabilistic backtracking trajectories of larval yellow perch (particle) dispersal prior to their collection with knowledge of larval age (i.e., days post-hatch from otoliths) can be used to revise initial hatching locations that, in turn, 1) improve the consistency of our breeding subpopulation discrimination functions that are based on larvae and 2) alter (and thereby seemingly enhance) estimations of the relative contribution of individuals from each breeding subpopulation to the to the mixed population of (age-0) juveniles in late summer, which is a strong predictor of future population size at the time individuals recruit to the fishery at age-2 [34–35]. In so doing, we use a range of hatch-location assignment thresholds to test the sensitivity of both natural tagging approaches.

## Materials and Methods

### Study species and system

Lake Erie (USA-Canada) is a part of the Laurentian Great Lakes system, and is warmer, shallower (mean depth of western basin = 7.4 m), and more productive than the other Great Lakes [36]. The western basin is a hydrodynamically active system, characterized by inflows from the Detroit and Maumee Rivers [37], large-scale circulation primarily driven by the Detroit River inflow and basin-wide winds [33], and the formation of a turbid river plume driven by the Maumee River inflow [27].

The yellow perch population in Lake Erie’s western basin contributes to economically important recreational and commercial fisheries [35] and appears to be supported by multiple discrete local breeding subpopulations [18, 27, 38–39]. Yellow perch larvae hatch at ∼5 mm total length (TL), are provided no parental care, and spend 30–35 d in the water column feeding on zooplankton before becoming demersal as juveniles at 20–25 mm total length (TL) [34, 40–41]. While the mixing of western basin north-shore and south-shore breeding subpopulations (i.e., potential stocks) is known to occur during the juvenile stage [27, 42], mixing of larvae has not been investigated. However, because yellow perch larvae are weak swimmers until ∼9.5 mm TL [43], we suspect that mixing of breeding subpopulations occurs during the larval stage, owing to the observed mixing of the Detroit River (north shore) and Maumee River (south shore) water masses during the larval production period [37, 44].

### Field collections

**Fig 1:**
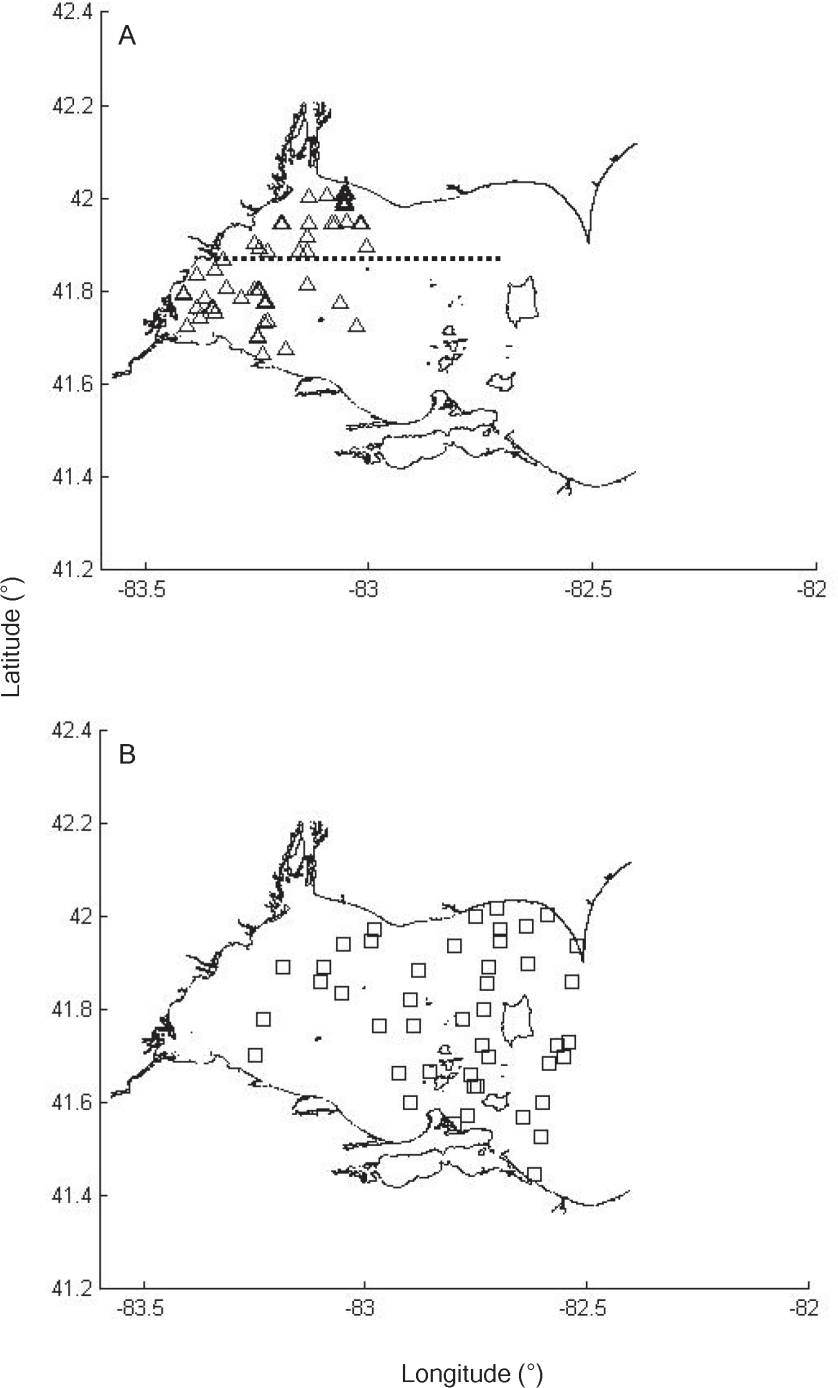
Larval (a) and juvenile (b) yellow perch collection sites during 2006 and 2007 in western Lake Erie. The dotted horizontal line in (a) marks the boundary between the north shore (NS) and south shore (SS).

Larval yellow perch were collected weekly during late April through June 2006 and 2007 at up to 12 sites near the north shore (NS) and south shore (SS) of Lake Erie’s western basin (Fig. 1A, see also [27]). For the purpose of this study, a line at latitude 41.81° N (approximately the northernmost extent of the Maumee River plume) was used to divide the NS and SS spawning grounds. All larvae were collected with paired bongo nets (1 m diameter; 500 μm mesh and one with 1000 μm mesh) towed ∼2 m from the bottom of the lake to the surface (∼8 min tows). All larvae were euthanized with a lethal dose of tricaine methanesulfonate, then preserved in 100% ethanol until identification and processing. In total, 242 larvae were captured during 2006 (n = 151 in NS; n = 91 in SS) and a total of 364 larvae were captured during 2007 (n = 283 in NS; n = 81 in SS).

Bottom-trawling surveys (10.7 m headrope, 13 mm cod-end liner, 3 km h^−1^ tow speed) for juvenile (age-0) yellow perch were conducted by the Ohio Department of Natural Resources-Division of Wildlife and Ontario Ministry of Natural Resources during late August, 2006 and 2007, at 71 and 73 sites, respectively (Fig. 1B, see also [27]), with trawl-site selection based on a stratified (by depth), random design [35]. Juveniles were euthanized with a lethal dose of tricaine methanesulfonate and kept frozen until processing. We processed 119 and 167 juveniles during 2006 and 2007, respectively, with the number of individuals processed from each site determined proportionally from trawl catch per unit effort data.

During 2007, water samples were also collected at each site from 1–2 m depth for trace-elemental analysis. The samples were filtered through 0.45 μm nylon filters, acidified with nitric acid (1% of the total volume of water; 0.5 mL acid to 20 mL water), and analyzed using ICP-MS.

### Ethics Statement

Larval and juvenile yellow perch were collected from public areas (Fig. 1) under the authority and with the assistance of the Ohio Department of Natural Resources-Division of Wildlife (Sandusky, OH) and the Ontario Ministry of Natural Resources (Wheatley, ON). No protected species were sampled. All work was approved by The Ohio State University’s Institutional Animal Care and Use Committee (Protocol number 2008A0056).

### Larval otolith extraction and hatch date determination

Otoliths were used to estimate the hatch date of each larva collected in each breeding area. Briefly, both sagittal otoliths were removed from larvae with glass probes under a Class 100 clean hood, with the otolith used for aging being mounted to a glass microscope slide with Crystalbond 509 thermoplastic cement (Structure Probe, Inc., West Chester, PA) and otolith used in micro-elemental analysis mounted to a petrographic glass slide using double-sided tape. All glassware were acid-washed prior to use, with all otoliths sonicated and further cleaned in ultra-pure deionized water (see [18, 27] for more details on the processing and cleaning process).

To determine the hatch date of each larva, we first identified the hatch check [27] and then counted post-hatch daily rings to the longest otolith edge, using ImagePro imaging software (Media Cybernetics, Inc., Rockville, MD) and a Nikon E200 compound microscope (100x and 50x magnification, oil immersion, Nikon Inc., Melville, NY). Otolith ages from larvae < 25 d old were determined from a single count, as previous research conducted with Lake Erie yellow perch showed that single ring counts are reliable for yellow perch of less than this age [34]. For larvae > 25 d of age, at least one additional blind count was conducted, with additional counts being performed as needed [27]. With knowledge of the each larva’s post-hatch age and day of collection, we could determine hatch date.

### Predicting larval dispersal through hydrodynamic backtracking

#### Water circulation model

A hydrodynamic model was used to simulate the currents and temperatures in Lake Erie, providing the three-dimensional physical predictions necessary for tracking individual particles (larvae). Our model is based on the Princeton Ocean Model platform [45], which solves the hydrostatic, three-dimensional primitive equations in a second-order finite difference framework. The model used the Smagorinsky parameterization for the horizontal diffusion (coefficient of 0.1) and the Mellor-Yamada level 2.5 turbulence closure scheme in the vertical direction. Our model was calibrated and validated for Lake Erie using eight National Ocean Service (NOS) water level gauges along the shoreline, current measurements in each basin, and lake surface temperature in each basin [22]. It currently runs in real-time as part of the NOAA Great Lakes Coastal Forecasting System (GLCFS; [46]), where hourly observations of wind speed/direction, cloud cover, air temperature, and dew point temperature are used to compute three-dimensional currents, temperature, water level, and waves on a 2 km resolution unstructured grid (21 vertical sigma levels).

#### Particle backtracking

Hourly output from the real-time GLCFS was used to drive a Lagrangian particle transport model to simulate passive trajectories of individual larva in western Lake Erie. The latitude and longitude where each larva was collected (see above) and its post-hatch age from otolith ring counts [27] were used to set the particle-tracking model’s initial parameters. The particle-tracking model used a 2^nd^-order Lagrangian scheme [47] to simulate passive, neutrally-buoyant particle movement in three dimensions. We assumed the larvae to be passive, neutrally-buoyant particles based on their small size, weak swimming ability, and positive photacticity [40, 43]. The Smagorinksy parameterization was used for horizontal diffusion (coefficient of 0.005), based on previous calibrations [44], and a random-walk approach was used for vertical diffusion (0.0005 m^2^ s^−1^).

In each simulation, a group of particles (n = 5,000) was initiated at the capture location of each larva and spread over a 5 m radius; the pathways taken by these particles were used to create a probability distribution for the pre-capture dispersal path of the larva captured at that location. Water-current uncertainties and variability were accounted for by the calibrated diffusion coefficients/schemes, as described above, as well as the defined particle patch. Simulations were performed backward in time, starting at the time of capture and proceeding back to the estimated hatching day. Daily location and temperature were recorded for each particle, delineating the backtracked pathway from capture to hatch location for each larva. In addition, a grid-based probability of daily larva location was computed for each modeled grid cell by accounting for the percentage of 5,000 particles present within the cell at any given time. In this sense, the spatial distribution of a larva’s location and the point of highest probability could be determined for each day (S1 Appendix).

With knowledge of the probability that a larva was in any given cell on any given day since hatching, we determined the most likely hatching location (NS or SS) by summing the probabilities of potential hatch cells in each location (e.g., NS probabilities were summed for cells above 41.81° N). Doing so allowed us to test how certainty in hatch location (60, 70, 80, and 90% certainty thresholds) could influence subpopulation discrimination abilities and juvenile classifications (using methods describe below). Output from these analyses also were compared to the “Best Estimate”, which used the single most probable hatch cell to assign larvae to the NS or SS without consideration of a certainty threshold.

### Breeding subpopulation discrimination and juvenile classification

For both the genetics and otolith microchemistry described below, we first conducted all analyses without using backtracking (i.e., we assumed larvae originated in their capture location). These initial larval assignments served as our null condition. We then repeated all analyses, using backtracking predictions to revise initial larval hatching locations where appropriate (per above). For genetic analyses, we first used larvae of all size classes in analyses. Afterwards, we repeated all analyses with a subset of larvae that only included individuals < 8 mm TL, which are likely to be primarily passively dispersed [43, 48]. We also only included herein loci in Hardy-Weinberg equilibrium (7 of the 12 loci, see below), with analyses that used data from all 12 loci being found in S4 Appendix. For otolith microchemical analyses, we included all larvae in analyses due to smaller initial sample sizes.

#### Microsatellite extraction and genotyping

DNA was recovered from tissue samples using a plate-based extraction method [49]. Extracted larval DNA samples were re-suspended in 50 μL of Tris–EDTA buffer (10 mM Tris, 1.0 mM EDTA, pH 8.0), whereas juvenile DNA was re-suspended in 100 μL of the same buffer.

Each individual was genotyped at a total of 12 microsatellite loci [50]. PCR amplification was performed in 25 μL reactions with the following components: 1.5 μL of template DNA; 2.5 μL 10x PCR buffer; 2.5 μL of MgCl_2_ (25 mM); 0.3 μL of dNTPs (50 μM of each); 0.2 μL (0.5 μM) of dye-labeled primer; 0.2 μL (0.5 μM) of the reverse primer; and 0.10 U Taq polymerase (Applied Biosystems, Foster City, CA). PCR conditions were as follows: initial denaturation at 94°C for 2 min, followed by 35 to 40 cycles of denaturing at 94°C for 15 s; annealing at various temperatures for 30 s (following [50]); extension at 72°C for 30 s; and a final extension of 72°C for 10 min. Microsatellite allele sizes were determined using a LI-COR 4300 DNA analyzer (Lincoln, NE) and scored using GeneImage IR 4.05 (Scanalytics Inc., Rockville, MD).

#### Larval population genetic structure

First, Fisher’s exact tests for Hardy-Weinberg Equilibrium (HWE) were performed using Arlequin (v3.5.1.2; [51]). The α-level of each test was adjusted by dividing by the number of tests conducted (i.e., 12 tests, one per locus). Loci were considered unreliable and removed from subsequent analyses, if consistent violations of HWE occurred at the same locus across both subpopulations or within a subpopulation during both years. Second, tests for linkage disequilibrium were run for all pairs of loci in all larval groups (and age-0 juveniles) using Genepop (version 4.0.7; [52]). Third, F_ST_ estimates were calculated to assess genetic differentiation within a year between NS and SS subpopulations using Genepop (version 4.0.7, [52]; following [53]). Lastly, we used discriminant analysis of principal components (DAPC) in the R package ‘adegenet’ (version 1.3–9.2; [54–55]) to explore genetic structure (spatial data based on capture or hatch location was not included in our analysis). DAPC first transforms the data using principal components analysis, then uses k-means clustering to define clusters that maximize the variation among groups (based on the lowest Bayesian Information Criterion).

In describing larval population genetic structure, we were not concerned with identifying population substructure *per se*. Instead, we sought to identify whether at least a minimal level of structure existed, and then using this weak structure to develop assignment and discrimination functions. Previous studies have demonstrated that the most common assignment tests are robust to low differentiation among subpopulations (e.g., low F_ST_) and violations of assumptions (e.g., HWE; [56–57]).

#### Larval self-assignment with microsatellites

We used GENECLASS 2.0 [58] to conduct a rank-based self-assignment genotype test during both years [59] that was based on microsatellite information from larvae from both subpopulations. It uses a bootstrapping approach wherein each individual larva is removed from the analysis (one at a time) and subsequently treated as an “unknown” larva that is then assigned to NS or SS subpopulations, based on the genetics of all other individuals [59]. By determining the percent of larvae successfully assigned back to their NS or SS collection location (i.e., null larval assignment, no backtracking) or hatch site (i.e., after backtracking revision), as well as by exploring posterior probabilities of assignment for each individual, reliability in assignments for each subpopulation could be assessed.

#### Juvenile classification with microsatellites

Microsatellite information from both larvae and juveniles was used to quantify both breeding subpopulation’s relative contribution of juvenile recruits to the mixed population during both years. To do so, we used GENECLASS 2.0 to assign each unknown-origin juvenile to either the NS or SS breeding subpopulation. These determinations were made using the null larval assignments (i.e., not revising larval hatch locations using backtracking) and revised larval assignments (i.e., accounting for pre-capture dispersal using backtracking). Our analysis consisted of a two-step procedure [60]. First, we performed a Bayesian assignment [61] with Monte Carlo re-sampling using Paetkau et al.’s [62] simulation algorithm (10,000 simulated individuals at an assignment threshold of P = 0.05). The Bayesian analysis allowed us to exclude unknown-origin juveniles with less than a 30% of belonging to either one of our focal breeding subpopulations (NS or SS), as the potential for contributions from other local breeding subpopulations exists in Lake Erie [18]. Second, we classified remaining juveniles to the NS or SS, using a rank-based genotype assignment (frequency method; [59]). In so doing, we considered successful ranked-based assignments to be those with probability of 70% or higher of belonging to one of the two breeding subpopulations (hence, the second group assignment probability would be 30% or lower). Failed assignments (i.e., unknown origin) were those with likelihood between 30% and 70%.

#### Otolith microchemistry

We used laser ablation-inductively coupled plasma-mass spectrometry (LA-ICP-MS) to quantify trace-metal concentrations in each otolith not used for hatch date determination. An ICP-MS (model X7, Thermo Elemental, Franklin, MA) with a Continuum Surelite I solid-state ND-YAG laser (wavelength = 266 nm, maximum power = 20 mJ, pulse rate = 20 Hz, pulse rate = 4–6 ns) was used to measure isotopic masses, which then were used to determine lithium (Li), magnesium (Mg), manganese (Mn), zinc (Zn), strontium (Sr), barium (Ba), and lead (Pb) concentrations (see [18, 27] for more details). Calcium (Ca) was used as an internal standard, to correct for ablation-yield differences, and the occurrence of mass 120 (measured as ^120^Sn, tin isotope) in samples was used as a contamination indicator [18]. A glass standard (NIST 610) was analyzed twice before and after every 16 samples to correct for drift and estimate the precision (coefficient of variation) of the instrument between runs. The argon carrier gas was analyzed for 60 s before every sample to determine instrument background levels and to estimate the limits of detection (LOD) of every sample. For larvae, ablated transects spanned from the outer edge of the otolith, through the core, and to the opposite outer edge. We integrated the entire otolith except for 5–10 s at the outer edge to avoid tape contamination. For juveniles, we ablated the portion of the otolith that captured the larval period based on the mean otolith radius length of 28 d old larvae caught from two breeding locations. We integrated and analyzed this larval portion before, through, and after the core [27]. Transect data were integrated using ICP-MS PlasmaLab software (Thermo Electron, Waltham, MA) to quantify mean elemental concentrations.

To be included in final analyses, concentrations had to be above the LOD for 90% of the samples within a breeding area and the glass standard coefficient of variation had to be < 10.5% [18]. For 2006 and 2007, Sr and Ba met our LOD criteria. Elements that were significantly related to otolith radius based on the results of an analysis of covariance (ANCOVA) were detrended to remove the effect of fish size using the slope of the relationship between the elemental concentration and the otolith radius [18, 31].

#### Larval self-assignment and juvenile classification using otolith microchemistry

We used linear discriminant function analysis (LDA), quadratic discriminant function analysis (QDA), random forest analysis (RF), and neural network analysis (NN) in R [63] to test for self-assignment consistency within each year. The LDA and QDA analyses used jackknife procedures to classify larvae. RF and NN use machine-learning algorithms. Based on the near-identical results across all methods for larval self-assignment, we used only LDA in R to quantify the contribution of both breeding subpopulations to the mixed population of unknown-origin juvenile recruits within each year [64]. We used similar cutoffs as with the genetic assignments to assign juveniles (i.e., likelihoods > 70% were successfully assigned to a subpopulation, whereas likelihoods < 70% were considered to be failed assignments). For both the larval self-assignment tests and the juvenile assignments, used the same null larval capture-location assignments (no backtracking) and backtracking-revised hatching-location assignments as in the microsatellite analyses.

## Results

### Hatch location determination using backtracking

Backtracking data from both years led us to revise the initial assignment of numerous larvae, mostly shifting the larval hatching location from the SS to the NS (S1 Appendix). During 2006, 151 of 242 larvae (62%) were captured in the NS, with the remaining 91 larvae collected in the SS. Reenactment of pre-capture dispersal pathways by backtracking, however, suggested that the hatching location of > 50 larvae should have been the NS instead of the SS (Best Assignment, using all larvae: 84% NS). Including the < 8 mm TL cutoff and more stringent certainty criteria regarding the confidence in our backtracked hatch locations further increased the percentage of larvae predicted to have hatched in the NS, but also simultaneously reduced the number of individuals included in subsequent analyses (no backtracking: 64% NS, total n = 190; Best assignment: 88% NS, n = 165; 60% certainty: 87% NS, n = 165; 70% certainty: 88% NS, n = 163; 80% certainty: 89% NS, n = 160; 90% certainty: 91% NS, n = 149). During 2007, 283 of 364 larvae (78%) were captured in the NS, with the remaining 81 larvae collected in the SS. Consideration of backtracked dispersal pathways prior to capture again shifted the initial assignment of hatch location more strongly toward larvae originating from the NS (Best assignment including all larvae: 88% NS). Including the < 8 mm TL cutoff and increasing our level of confidence in the backtracking data again increased the percentage of larvae predicted to have originated in the NS and reduced the number of larvae that could be used to build functions to assign juveniles (no backtracking: 84% NS, total n = 247; Best assignment: 91% NS, n = 242; 60% certainty: 91% NS, n = 240; 70% certainty: 91% NS, n = 240; 80% certainty: 93% NS, n = 236; 90% certainty: 98% NS, n = 224).

### Larval population genetic structure

For larvae captured during 2006, two of the NS loci and five of the SS loci (out of 12 for each subpopulation) deviated significantly from HWE. One locus deviated significantly from HWE across both subpopulations during 2006 (Table 1). During 2007, 6 of the 12 NS loci and 5 of the 12 SS loci significantly deviated from HWE in larvae from both the NS and SS. Five loci deviated significantly from HWE across both subpopulations during 2007 (Table 1). Because 5 of 12 loci (loci YP78, YP96, YP60, YP65, and YP49) significantly deviated from HWE in either both years within a subpopulation or in both subpopulations within a year, we removed these loci from subsequent analyses, unless noted otherwise. No evidence of linkage disequilibrium was found in either year. See S2 Appendix for genetic diversity indices based on larvae < 8 mm TL and including only the seven loci in HWE.

**Table 1:** Number of genotypes (N), alleles (N_A_), and observed (H_O_) and expected (H_E_) heterozygosity for the 12 microsatellite loci (Li et al. 2007) used to genotype larval yellow perch (YP) collected in north-shore (NS) and south-shore (SS) waters of Lake Erie’s western basin during 2006 (N_NS larvae_ = 151, N_SS larvae_ = 91) and 2007 (N_NS larvae_ = 283, N_NS larvae_ = 81).

		Locus											
Groups		YP85	YP78	YP41	YP109	YP55	YP110	YP96	YP60	YP65	YP49	YP81	YP99
NS06	N	137	**140**	141	116	123	129	133	140	147	**142**	135	125
	N_A_	17	**13**	6	24	9	9	10	6	13	**10**	6	13
	H_O_	0.79	**0.89**	0.67	0.82	0.56	0.12	0.67	0.54	0.64	**0.77**	0.67	0.86
	H_E_	0.84	**0.84**	0.58	0.93	0.50	0.12	0.50	0.44	0.57	**0.65**	0.54	0.86
SS06	N	**76**	**78**	87	**57**	82	74	84	79	**79**	83	**76**	74
	N_A_	**16**	**11**	8	**20**	7	5	6	9	**11**	7	**16**	12
	H_O_	**0.61**	**0.73**	0.69	**0.79**	0.57	0.11	0.60	0.65	**0.61**	0.75	**0.51**	0.76
	H_E_	**0.81**	**0.84**	0.58	**0.94**	0.53	0.15	0.51	0.55	**0.60**	0.62	**0.73**	0.85
NS07	N	267	**278**	269	**271**	280	260	**282**	**269**	**266**	**274**	272	272
	N_A_	22	**15**	7	**30**	7	9	**13**	**9**	**10**	**12**	6	15
	H_O_	0.75	**0.89**	0.64	**0.80**	0.61	0.12	**0.74**	**0.69**	**0.82**	**0.97**	0.60	0.87
	H_E_	0.78	**0.85**	0.60	**0.94**	0.53	0.12	**0.58**	**0.54**	**0.62**	**0.67**	0.59	0.86
SS07	N	61	**78**	81	65	80	77	**79**	**66**	**76**	**67**	74	68
	N_A_	18	**12**	6	22	6	7	**6**	**6**	**8**	**6**	5	12
	H_O_	0.92	**0.99**	0.60	0.97	0.68	0.10	**0.72**	**0.82**	**0.79**	**0.87**	0.64	0.93
	H_E_	0.87	**0.84**	0.57	0.94	0.55	0.13	**0.51**	**0.58**	**0.58**	**0.64**	0.55	0.84

Groups are denoted by collection location (NS, SS) followed by the year of collection (06, 07). Note: Data in bold denotes deviations from HWE (following Bonferroni correction).

Pairwise F_ST_ values, based only on the seven loci, indicated only weak genetic differentiation between the NS and SS subpopulations during 2006 and 2007 (Table 2). Using backtracking information to improve the certainty of the larval hatch location caused a ∼3-fold (Best; i.e., > 50% certainty in hatching location) to > 5-fold (90% certainty in hatching location) increase in the F_ST_ value (relative to the null condition; i.e., no backtracking) during 2006, although a similar increase was not evident during 2007 (Table 2). Conducting the same analyses with only larvae < 8 mm TL (i.e., those most likely to drift passively) caused F_ST_ values to generally increase during both years relative to when all larvae were included in the analysis. Further, increasing confidence in the hatching location of larvae (those < 8 mm TL) with backtracking generally led to a 3-fold to 4-fold increase in the F_ST_ value over the null condition (Table 2).

**Table 2:** F_ST_ values between larvae collected in north-shore (NS) and south-shore (SS) waters of western Lake Erie during 2006 and 2007 based on seven microsatellite loci, with all larvae included (top) and only larvae < 8 mm total length included (i.e., those most likely to be passively dispersed; bottom).

	2006	2007
	n, F_ST_	n, F_ST_
None	242, 0.0083	364, 0.0016
Best	213, 0.024	355, 0.0017
60%	211, 0.028	347, 0.0018
70%	205, 0.035	340, 0.0020
80%	197, 0.038	332, 0.015
90%	186, 0.044	316, 0.022
None & < 8 mm	190, 0.010	247, 0.0016
Best & < 8 mm	165, 0.039	242, 0.0051
60% & < 8 mm	165, 0.038	240, 0.0049
70% & < 8 mm	163, 0.041	240, 0.0049
80% & < 8 mm	160, 0.042	236, 0.0010
90% & < 8 mm	149, 0.045	224, 0.027

Sample sizes (n) are included. Rows indicate confidence in hatch-location assignment: None: null assignments based on capture location; Best: assigned to single most likely hatching location (SS or NS) based on backtracking; 60%: assigned to hatching location with at least 60% confidence; 70%: assigned to hatching location with at least 70% confidence; 80%: assigned to hatching location with at least 80% confidence; 90%: assigned to hatching location with at least 90% confidence.

DAPC also provided evidence for genetic differentiation, using seven microsatellite loci. Near equal support for two to five genetic clusters was found during 2006 using the full set of larvae (S3 Appendix). Using only larvae that were < 8 mm TL and met the 90% hatch-location certainty criterion gave similar results (S3 Appendix). For the full set of 2007 larvae, DAPC found the strongest support for four clusters, with three to seven clusters similarly supported (S3 Appendix). Using only 2007 larvae that were < 8 mm TL and with a 90% hatch location certainty gave similar results (S3 Appendix). Because our interest was in discriminating between subpopulations from the SS and NS for this application, we plotted two clusters for each dataset against the first discriminant function to illustrate the improved consistency of each cluster after using the backtracking procedure to revise initial group membership (i.e., we compared cluster assignments of all larvae to assignments of larvae for which hatch location was most certain; S3 Appendix).

### Larval self-assignment accuracy (microsatellites)

**Fig 2:**
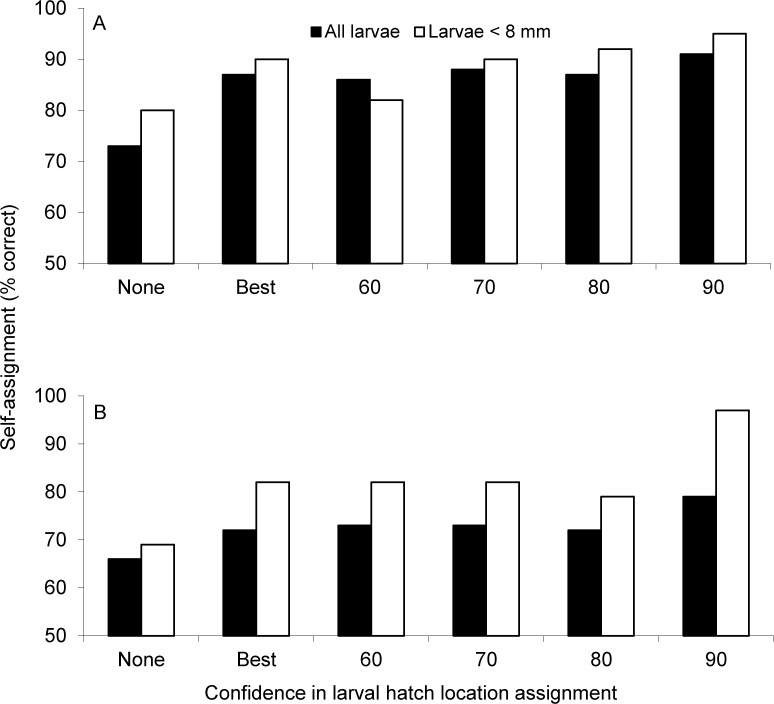
Self-assignment results based on microsatellite DNA data (seven loci) for larval yellow perch collected in Lake Erie’s western basin during a) 2006 and b) 2007. For sample sizes, see Table 2. Confidence in hatching locations is as follows: None = null assignments based on capture location (i.e., no backtracking used); Best = larvae assigned to single most likely hatching location (SS or NS) based on backtracking; and 60, 70, 80, 90 = larvae assigned to hatching location after backtracking revision with 60, 70, 80, 90% levels of certainty in hatching origin, respectively.

Using backtracking to correct for pre-capture dispersal and only using small (< 8 mm TL) larvae that are likely to drift passively improved larval self-assignment accuracy. During 2006, self-assignment success of larvae increased from ∼73% without backtracking (i.e., initial hatching-location assignment based on larval capture location) to 85% to 90% with consideration of backtracking at all levels of larval hatching-location certainty when no size cutoff was used (Fig. 2A, black bars). Implementing the 8 mm TL size cutoff tended to increase larval assignment success by a small percentage, with >90% self-assignment success being achieved when the most stringent (90%) backtracking certainty was used (Fig. 2A, white bars). During 2007 (Fig. 2B), the pattern was similar, although backtracking offered more modest gains (< 12%) in assignment success (from ∼65% success to ∼78% success). However, when the 8 mm TL size cutoff was implemented, leaving only larvae that are likely to drift passively [43], self-assignment accuracy rose to >80% for most of the lower levels of certainty in larval hatching location, but increased to 97% when the 90% certainty criterion in backtracked hatching location was implemented (Fig. 2B).

### Classification of unknown-origin juveniles (microsatellites)

**Fig 3:**
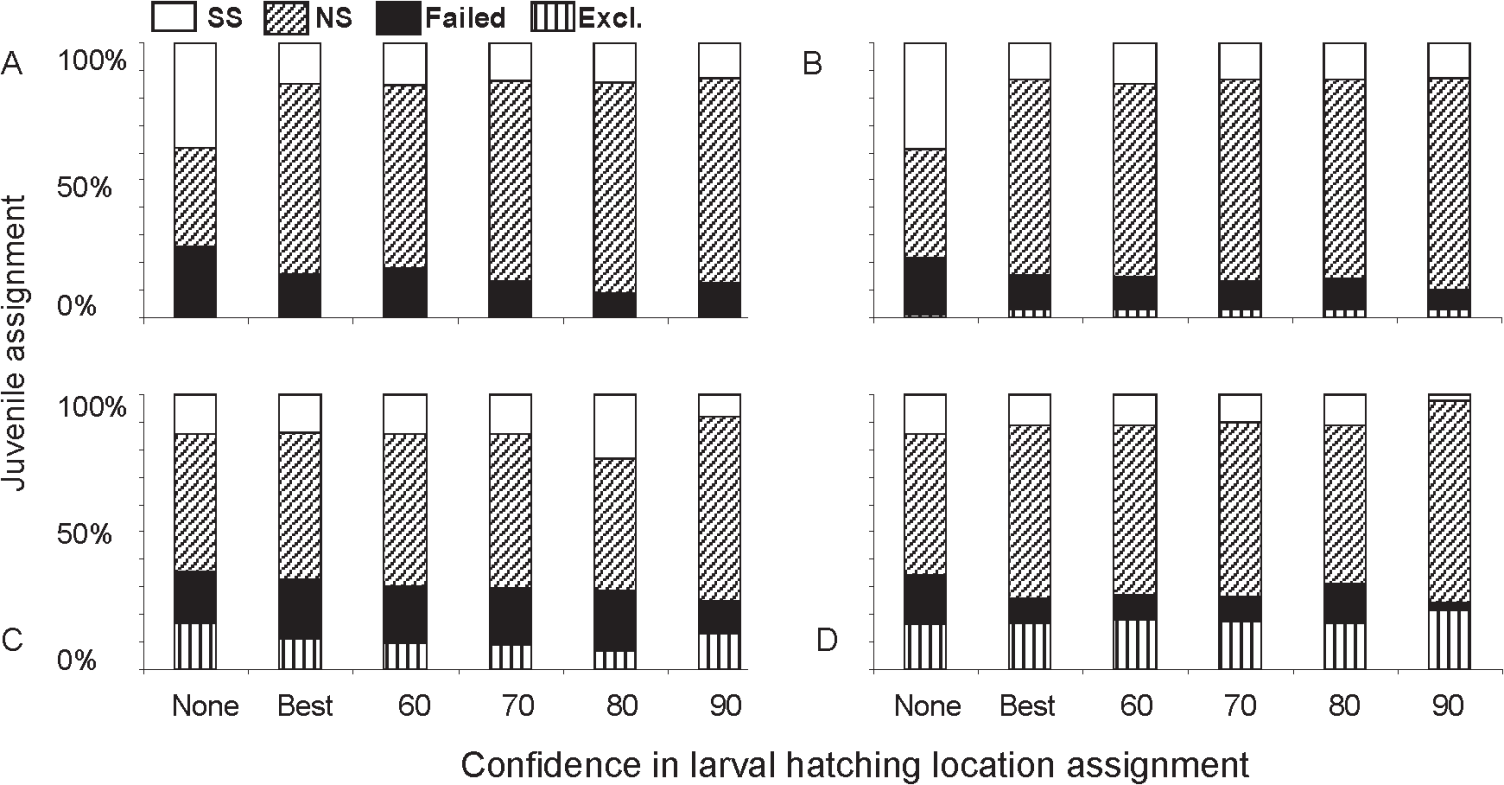
Predicted origins of juvenile yellow perch collected in open waters of western Lake Erie using microsatellites (seven loci) from larvae collected during 2006 (a: all larvae; b: only larvae < 8 mm total length, TL) and 2007 (c: all larvae; d: only larvae < 8 mm TL). A total of N = 119 and N = 167 juveniles were analyzed during 2006 and 2007. Juveniles with < 30% likelihood of originating within a population were “excluded” (i.e., they were not included in the analysis); juveniles with a 30 to 70% likelihood were considered “failed,” (i.e., we had little confidence in their hatching-location assignment); and juveniles with a probability > 70% were assigned to the NS or SS breeding population with high confidence. Certainty in hatching locations of *larvae* used to develop classification functions was as follows: None = null assignments based on capture location (i.e., no backtracking used); Best = larvae assigned to single most likely hatching location (SS or NS) based on backtracking; and 60, 70, 80, 90 = larvae assigned to hatching location after backtracking revision with 60, 70, 80, 90% levels of certainty in hatching origin, respectively.

Improved classification accuracies resulting from consideration of corrected larval hatching locations (via backtracking) and implementation of an 8 mm TL size cutoff (see Fig. 2) both improved our ability to determine the origins of juvenile recruits captured in a mixed population during August 2006 and 2007 and altered each breeding subpopulation’s predicted contribution to it. Some individuals (0% to 20%, depending on the analysis) likely did not emanate from either of our breeding populations, and therefore were excluded from analysis (Fig. 3). However, of those that were viable for classification, the percentage of failed assignments decreased by ∼2-fold with consideration of both backtracking and the 8 mm TL cutoff during both years, with the reduction in failed assignments being far greater when the 90% certainty level in backtracked hatch location was used (Fig. 3). In addition, during both years, the percentage of juveniles classified to NS tended increased, with the percentage assigned to the SS concomitantly decreasing. During 2006, the percentage of juveniles predicted to emanate in the NS increased by ∼20% to ∼30% with consideration of backtracked hatching location, whereas the increase on the order of ∼10% during 2007.

### Larval self-assignment accuracy (otolith microchemistry)

Mean otolith Sr concentrations in larvae differed strongly between the NS and SS breeding subpopulations during both years (2006: SS: 847±216 μg g^−1^, NS: 443±55 μg g^−1^; 2007: SS: 843±239 μg g^−1^, NS: 554±103 μg g^−1^), which reflected differences in water chemistry (Sr:Ca ratios) between breeding locations (2006: SS: 0.0021, NS: 0.0011; 2007: SS: 0.0021, NS: 0.0014). Mean Ba concentrations in both larval yellow perch otoliths and the ambient water also differed between breeding locations during both years (2006: SS: 30±24 μg g^−1^; NS: 99±37 μg g^−1^; 2007: SS: 50±26 μg g^−1^; NS: 125±213 μg g^−1^; Ba:Ca ratios: 2006: SS: 0.000075; NS: 0.00025; 2007: SS: 0.00012; NS: 0.00031 Ba:Ca). During 2007, however, Ba exhibited a significant interaction between subpopulation and otolith radius (*F*_1,111_ = 30.25, *P* < 0.001) and could not used for discrimination. Because otolith size was significantly related to Sr concentrations in both years and Ba in 2006 (2006 Ba: *F*_1,36_ = 16.24, *P* < 0.001; 2006 Sr: *F*_1,36_ = 128.14, *P* < 0.001; 2007 Sr: *F*_1,111_ = 128.14, *P* < 0.001), we detrended the data using slope estimates of −0.340 (2006 Ba), −0.00134 (2006 Sr), and −0.00161 (2007 Sr).

**Fig 4:**
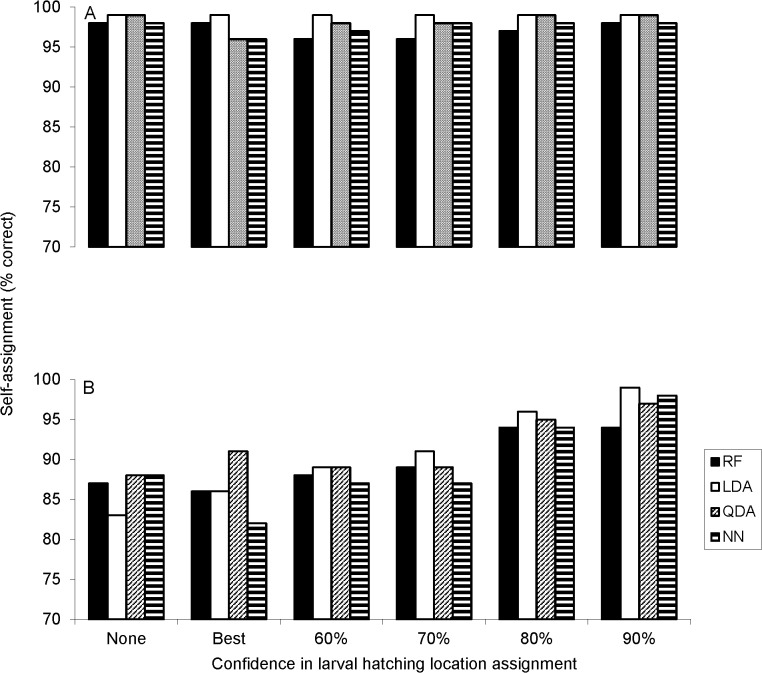
Larval yellow perch self-assignment results using a Random Forest (RF), Linear Discriminant Function Analysis (LDA), Quadratic Discriminant Function Analysis (QDA), and Neural Network (NN) based on otolith microchemistry data for a) 2006 and b) 2007. A total of N = 47 and N = 71 larvae were analyzed in 2006 and 2007. Confidence in hatching locations is as follows: None = null assignments based on capture location (i.e., no backtracking used); Best = larvae assigned to single most likely hatching location (SS or NS) based on backtracking; and 60, 70, 80, 90 = larvae assigned to hatching location after backtracking revision with 60, 70, 80, 90% levels of certainty in hatching origin, respectively.

Self-assignment accuracy of larvae, based otolith micro-elemental concentrations, was high during both years, with the value of backtracking differing between years. Owing to otolith Sr being a near-perfect discriminator in 2006, larval self-assignment success across all multivariate tests was high (> 98%) even without consideration of backtracking, and remained similarly high with each backtracking scenario (Fig. 4A). During 2007, assignment success across all multivariate tests was slightly lower (83–87%) without consideration of backtracking results, but increased to > 95% (up to 98%) with the most stringent backtracking criterion (90% certainly in larval hatch location, Fig. 4B).

### Classification of unknown-origin juveniles (otolith microchemistry)

**Fig 5:**
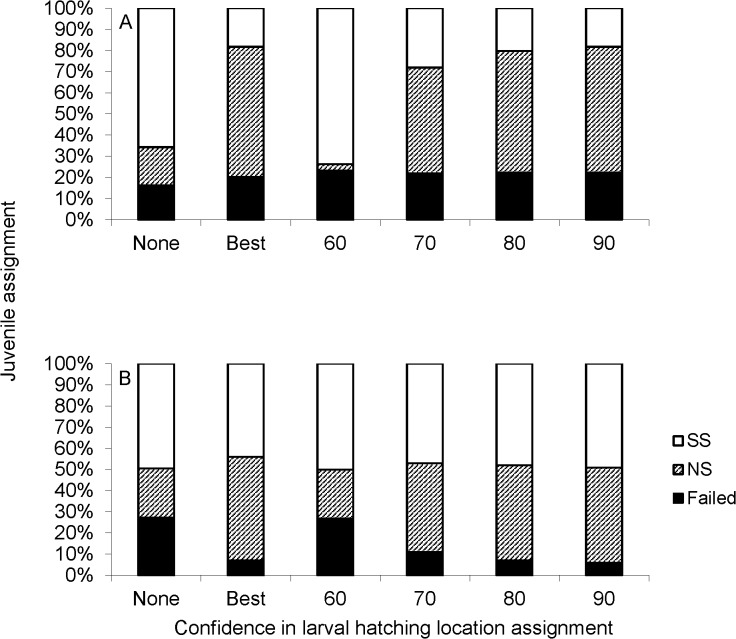
Predicted origins of juvenile yellow perch collected in open waters of western Lake Erie using otolith microchemistry from larvae collected in north-shore (NS) and south-shore (SS) water during a) 2006 and b) 2007. A total of N = 98 juveniles were analyzed each year. Juveniles with < 70% likelihood of originating within a population were consider “failed” and juveniles with a probability > 70% were assigned to NS or SS. Certainty in hatching locations of *larvae* used to develop classification functions was as follows: None = null assignments based on capture location (i.e., no backtracking used); Best = larvae assigned to single most likely hatching location (SS or NS) based on backtracking; and 60, 70, 80, 90 = larvae assigned to hatching location after backtracking revision with 60, 70, 80, 90% levels of certainty in hatching origin, respectively.

Similar to the assignments of juveniles based on microsatellite information (see Fig. 3), backtracking showed potential to improve the use of otolith microchemistry for identifying the hatching locations of juvenile recruits. While backtracking did not reduce the percentage of failed assignments (i.e., < 70% classification accuracy) during 2006 (Fig. 5A), the percentage of failed assignments declined from ∼ 25% to < 5% during 2007 (Fig. 5B). In addition, consideration of backtracked hatch locations of larvae used to develop classification functions altered the relative contributions of juveniles from both subpopulations during both years. The proportion of the total fish assigned to the NS increased in both years relative to the null (no backtracking) scenario. During 2006, this increase was >50% for the Best and 90% backtracking-certainty scenarios (Fig. 5A). This increase in individuals assigned to NS corresponded to a decrease in those assigned to SS. During 2007, the percentage of juveniles predicted to originate in the NS doubled with consideration of backtracking (from ∼20% without it to ∼40% with it; Fig. 5B). However, during 2007, the percentage of the total fish assigned to SS remained fairly constant (∼45%). Rather, during 2007, the increase in individuals assigned to the NS corresponded to a decrease in the percentage of failed assignments (from ∼25% to < 5%; Fig. 5B).

## Discussion

Our results demonstrate that the use of particle backtracking approaches that account for dispersal history prior to collection can improve the ability of natural tagging approaches to discriminate among breeding subpopulations (i.e., potential stocks). In addition, the general backtracking approach used herein is valuable in that it allows for detection and characterization of population structure despite physical mixing of individuals at a young age (e.g., in the case of western Lake Erie, transport of larvae from one hatching location to another by strong river- and wind-driven currents), which none of the classical population genetics methods can do without an unmixed control sample to train the analysis to identify strays. For both natural tagging approaches used herein (i.e., microsatellites and otolith micro-elemental composition), revision of larval hatch locations via particle backtracking simulations improved our ability to develop reliable classification functions breeding subpopulation discrimination. In our example, the use of backtracking improved the accuracy of assignment of larval yellow perch to their proper breeding subpopulation in western Lake Erie (NS or SS) by as much as 43% over the null condition (i.e., use of larval capture location without backtracking; Table 3). In addition, larval self-assignment accuracies tended to increase with increasing confidence in the backtracked pre-capture dispersal trajectories. Using backtracking to correct the hatch locations of larvae used to develop classification functions also generally caused F_ST_ values measured between breeding subpopulations to increase, suggesting that this method could assist efforts to quantify genetic structure in weakly differentiated stocks. An improved ability to discriminate between breeding subpopulations (using genetics or otolith microchemistry) would, in turn, be expected to benefit efforts to correctly identify the source origin of older individuals found in a mixed population. In our western Lake Erie application, the use of backtracking to revise some of the larval hatch locations caused the percentage of failed juvenile recruit classifications to decrease by half or greater (relative to the null, no-backtracking scenario; Table 3) and also caused us to revise the relative subpopulation contribution of juvenile recruits from the NS versus SS. The increase in the contribution of juvenile recruits from the NS with consideration of backtracking was as much as 45% of the total number of individuals (a 4x increase over initial estimated contributions from this subpopulation when backtracking was not used). As we explain below, changes as substantial might not only lead to a new understanding of population structuring and dynamics in an ecosystem, but also simultaneously benefit conservation or management efforts.

**Table 3:** Summary of the percentage of individuals correctly self-assigned (larvae) or successfully classified to a natal group (juveniles) without and with backtracking revision of initial larval assignments.

Microsatellite data	No backtracking	Backtracking
Larval assignment		
2006	80%	94%
2007	69%	97%
Juvenile classification		
2006	79%	90%
2007	66%	78%
Otolith microchemistry		
Larval assignment (LDA)		
2006	96%	98%
2007	83%	99%
Juvenile classification		
2006	82%	77%
2007	71%	92%

The backtracking column includes only the data using the 90% confidence threshold for larval hatching location assignment. The data also include only larvae < 8 mm TL.

### Application of particle backtracking to Lake Erie yellow perch

Specific to western Lake Erie yellow perch, contributions of juvenile recruits from the NS and SS regions have been previously documented in western Lake Erie using genetics [42] and otolith microchemistry [27], respectively. However, like many studies conducted in other aquatic ecosystems, these studies did not account for potential dispersal prior to larvae being collected for development of discrimination functions. Our consideration of passive dispersal during the larval stage (using backtracking) suggests that the NS subpopulation contributes more larvae and juvenile recruits to the open-lake population than has been previously reported (e.g., [42] as inclusion of backtracking increased the contribution of juvenile recruits from the NS by up to 60% and 108% during 2006 and 2007, respectively. Though direct comparisons to [27] would not be appropriate because that investigation compared relative recruitment rates of larvae residing inside versus outside of an open-lake river plume (Maumee River) that is generally only found in the SS of western Lake Erie and focused on larval habitat use rather than natal origin, our findings do suggest that a large proportion of the individuals that used this river plume as nursery habitat during the larval stage actually originated in the NS (49% and 47% of the larvae captured in 2006 and 2007, respectively). We suspect that many of these larvae were passively advected by strong Detroit River water currents from the NS into the SS prior to capture for discrimination purposes (the mean travel time is less than 21 hours; [65]), most likely at an early age when they are expected to have weak swimming abilities [43].

Recognition of the heightened importance of the NS to juvenile recruit production most certainly would be of interest to Lake Erie researchers and fisheries management agencies. Ecologists, for example, might be interested in learning more about the mechanisms that allow for maintenance of this weak genetic differentiation. While such a discussion is beyond the scope of this study, natal homing behavior and kin recognition/selective mating provide two reasonable hypotheses [66–67]. By contrast, Lake Erie agencies might wonder whether the NS larvae and subsequent juvenile recruits are originating from outside of Lake Erie proper, given that the current management plan for Lake Erie yellow perch does not consider the Detroit River – Lake St. Clair corridor as a contributing subpopulation [35]. The possibility certainly exists that the majority of NS larvae are produced outside of Lake Erie proper, which would have a profound effect on predictions of Lake Erie agency-derived yellow perch population size that in part depends on recruitment being dictated by in-lake spawning stock biomass [35]. Further, research conducted with Pacific salmonines has demonstrated that maintenance of a diverse “portfolio” of breeding subpopulations (i.e., stock diversity) can be critical to ensuring population viability during changing ecosystem conditions [68]. Given that Lake Erie has experienced large-scale ecosystem change during recent decades, owing to such factors as altered nutrient inputs [69, 70], invasive species [71], and climate change [72–73], we recommend investigations that use a backtracking approach in combination with one or more natural tagging approaches to determine whether larvae collected in the Detroit River – Lake St. Clair corridor can be discriminated from NS and SS larvae. If discrimination is possible, quantification of whether this outside source contributes recruits to the juvenile population, which is a strong predictor of future recruitment to the fishery at age-2 [35], seems a logical next step.

### Application of particle backtracking to other populations

Lake Erie yellow perch have life-history characteristics that are similar to many other freshwater and marine organisms. Such traits include the production of small propagules (i.e., eggs, larvae) that are subject to passive dispersal – for at least a period of time—by physical forces such as water circulation [74]. For this reason, hydrodynamic models have been used extensively to describe the dispersal trajectories of eggs and/or larvae in other fish populations, both freshwater [75] and marine [76], as well as aquatic invertebrates, including mollusks [77], crustaceans [78], and corals [79]. While this modeling historically has consisted of forecasting the post-spawning dispersal trajectories of eggs or larvae, efforts to hindcast dispersal histories of eggs or larvae prior to capture from a known collection (sampling) location have increased in recent years [21, 80].

Although our discussion thus far has focused primarily on aquatic organisms, we see great potential for our combined backtracking-natural tagging approach to also benefit our understanding of and the management of the dynamics of terrestrial organisms. Because many terrestrial plant and invertebrate animal species also have life stages that are vulnerable to passive dispersal by physical processes such as wind, atmospheric models have been used in a similar way as water circulation models to describe dispersal trajectories of a variety of terrestrial organisms. Admittedly, the bulk of this research has been conducted with terrestrial plants [81]; however, the application to understanding the dispersal dynamics of both invertebrate animals [10, 81–82] and microorganisms [83] is readily apparent. For example, Guichard et al. [11] used atmospheric models to hindcast moth dispersal patterns.

Relatedly, our backtracking approach also can help to identify which discrimination approaches are likely to be most useful in a given system. If backtracking results, for example, indicate that individuals from different, breeding (source) populations have been mixed for a significant amount of time prior to capture such that they had experienced similar natal or post-hatching environments, discrimination based on tools that depend on these environmental differences (e.g., otolith microchemical approaches that rely on differences in elemental or isotopic composition; approaches that use parasites or contaminants as discriminators) may need to be complemented or substituted with other approaches that do not depend on environmental differences among breeding locations (e.g., genetic approaches).

### Backtracking approaches: caveats and research needs

While our analyses conducted with Lake Erie yellow perch highlight the value of using a backtracking model to account for dispersal history in breeding-subpopulation discrimination studies, this approach is not a panacea. As we discuss below, many important assumptions must be made when developing the backtracking model and the use of this technique can increase the need for heightened sample collections.

A key decision that will need to be made is whether to include behavior (i.e., active movement) in the backtracking model. In our application, we did not include any larval behavior and assumed that larvae drifted passively. While this assumption of passive dispersal appears valid for the sizes of yellow perch larvae used in our primary analyses (< 8 mm TL; [43]), its validity would be expected to decline with increasing fish size as active movement behavior has been shown to increase with ontogeny and body size, owing to in a large part a heightened ability of individuals to swim horizontally with more power [43, 84]. Thus, incorporation of active movement behavior in predictive models of dispersal may become necessary at times. Indeed, previous research conducted on both invertebrates [85] and fishes [30, 48, 86] has shown that even slight active horizontal or vertical movement during the larval stage can affect dispersal patterns. Such an effect might underlie the reduced discrimination ability between NS and SS yellow perch breeding subpopulations when all sizes of larvae were used in our study relative to when only those larvae < 8 mm TL were used. Given this consideration, the use of backtracking approaches that assume passive dispersal may not be valid for some species or only during very brief periods of time, thus requiring behavior (active movement) to be included in the backtracking model.

A related consideration when using backtracking approaches concerns the legitimacy of the backtracking model itself. In terms of constructing the backtracking model, many important decisions must be made with respect on how to deal with vertical diffusion, account for turbulent effects, and how stochasticity is included in the model, among many other considerations. Such considerations can be critically important. For example, how the stochasticity due to turbulence is accounted for in the backtracking model can lead to strongly differing predictions [28]. Predicting advection in nearshore habitat where larvae may be most common can be particularly uncertain [87–88]. While much research continues to be conducted in this area, and manuals have been developed for developing robust predictive models of particle transport in aquatic ecosystems [29], proper calibration and validation of the physical model must be conducted, if it is to provide useful data [28, 89]. Our backtracking simulations conducted during 2006 and 2007 follow known hydrodynamic phenomena in the western basin [33, 44], which can explain the subsequent reassignment of larvae origination. Wind-driven currents in the western basin dictate the transport of Detroit and Maumee River waters, including the extent, flushing time, and degree of mixing. In other sysems, many quality hydrodynamic and atmospheric models already have been developed for non-ecological applications, which could readily be applied for ecological studies (e.g., the hydrodynamics model used here was developed for another purpose, and adapted to model the transport of larval fish). In turn, interdisciplinary collaborations between physical modelers and ecologists most certainly would save both time and money by allowing a pairing of expertise and providing cost-effective ways to estimate spatially and temporally explicit environmental parameters that are difficult to measure via non-modeling methods [21].

While we showed that backtracking can improve the performance of natural tags for breeding subpopulation discrimination, this benefit did not come without a cost. Specifically, we learned that, as more stringent criteria were implemented to increase confidence in larval hatching locations, the number of individuals included in the analyses declined. For example, during 2006, our sample sizes used in genetic analyses declined from 68 individuals in the SS subpopulation when backtracking was not considered to 21, 20, 19, and 8 individuals when confidence in our backtracking estimate larval hatching locations was increased to 60%, 70%, 80%, and 90%, respectively. As a result of this tradeoff, achieving high confidence in discrimination ability may leave insufficient numbers of individuals to perform robust (powerful) assignments of individuals to their source origin. Indeed, previous research has demonstrated that minimal sample sizes exist in the (training) data used to develop classification functions, with these samples sizes varying among natural tagging techniques. For example, a minimum of 15 individuals from each breeding subpopulation has been suggested for the use of statolith microchemical approaches [9]. Likewise, a minimum of sample size of 25–30 individuals per breeding population has been suggested for the use of genetics as a natural tag [90]. As a result, more effort may be required during the initial collection phase to ensure that enough individuals remain in the analyses, once initial assignments to a breeding subpopulation are corrected via backtracking. Unfortunately, the number of additional individuals that would need to be collected at each breeding location will vary as a function of the degree of mixing of individuals among subpopulations, which will be driven in large part by stochastic water movement (or wind) in relation to propagule (e.g., seeds/eggs, larvae) production from each breeding subpopulation that mixes. Because we find it unlikely that one could estimate *a priori* how many individuals at a specific breeding location emanated from another breeding location, we recommend the collection of as many individuals at a given location that is practical. For situations in which such analyses are likely to be conducted annually, we are optimistic that a target number of individuals needed from each breeding subpopulation would emerge with time.

## General Conclusions

Despite the growing use of particle backtracking and natural tagging approaches in both aquatic and terrestrial ecosystems, we are unaware of any previous study that has coupled these approaches in a way that we have done here. Given the improved discrimination capabilities that resulted from our use of particle backtracking in conjunction with microsatellite and otolith microchemical information in a western Lake Erie fish population, we strongly believe that an integrated particle backtracking-natural tagging approach that accounts for pre-collection dispersal history holds great potential to enhance our ability to address a wide range of ecological questions that applicable to many ecosystem types. Such applications include: 1) determination of metapopulation and genetic structure/dynamics; 2) identification of recruitment mechanisms and how they vary among breeding subpopulations; 3) exploration of the degree of connectivity among populations; 4) assessment of whether local breeding subpopulations have evolved a life history in response to predictable physical features [91]; and 5) quantification of which local breeding or nursery area(s) disproportionately contribute(s) recruits to the broader population. Such advancement in our understanding of the natural world undoubtedly could be used to benefit management and conservation efforts. For example, identification of important breeding subpopulations or nursery areas could lead to altered population-specific harvest quotas (e.g., increased or decreased harvest, depending on whether the focal species was valued or considered a nuisance) or area-specific protection measures (e.g., no-take zones; [92]). By contrast, breeding subpopulations or areas that are found to not be contributing recruits in a manner that is on par with production at earlier life stages might require the establishment of rehabilitation efforts [7]. Given all of these potential benefits to science, management, and conservation, we strongly encourage the use of integrated particle backtracking-natural tagging approaches, as well as research geared towards overcoming some of their known limitations.

## Supporting Information

S1 Appendix Larval dispersal plots based on backtracking model. (PDF)

S2 Appendix Genetic diversity indices. (PDF)

S3 Appendix DAPC results. (PDF)

S4 Appendix Larval assignment and juvenile classification based on 12 microsatellite loci. (PDF)
