# Supplementary material for: Particle Backtracking Improves Breeding Subpopulation Discrimination and Natal-Source Identification in Mixed Populations
Source: PLoS One. 2015 Mar 23;10(3):e0120752. doi: 10.1371/journal.pone.0120752 (PMC4370746; doi:10.1371/journal.pone.0120752)
Supplement: S1 Appendix — (PDF) [file pone.0120752.s001.pdf]

S1 Appendix Larval dispersal plots based on particle backtracking model

Plots of most probable larval dispersal pathways (Best assignment) in western Lake Erie during 2006 and 2007 based on particle backtracking model. Yellow perch larvae collected in the SS are marked in red. Larvae collected in the NS are marked in blue. The dotted horizontal lines mark 41.81° N. In the large majority of cases, when larvae were advected from one breeding region to the other, the movement was from the NS to the SS.

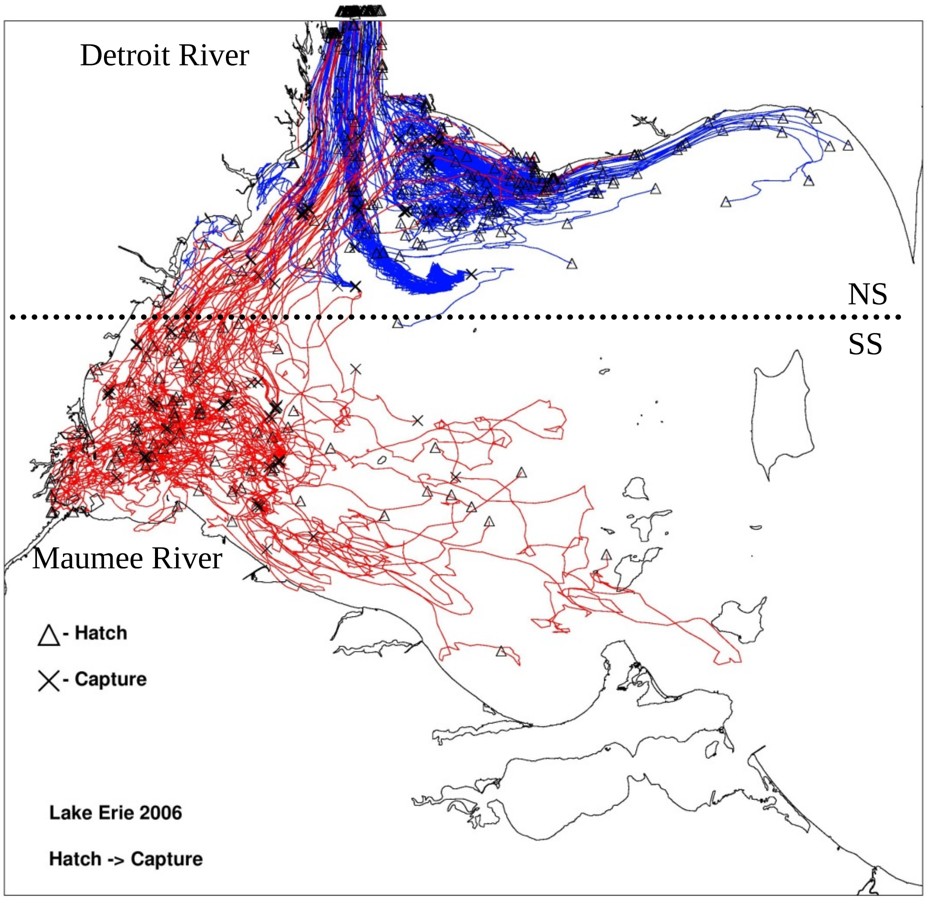

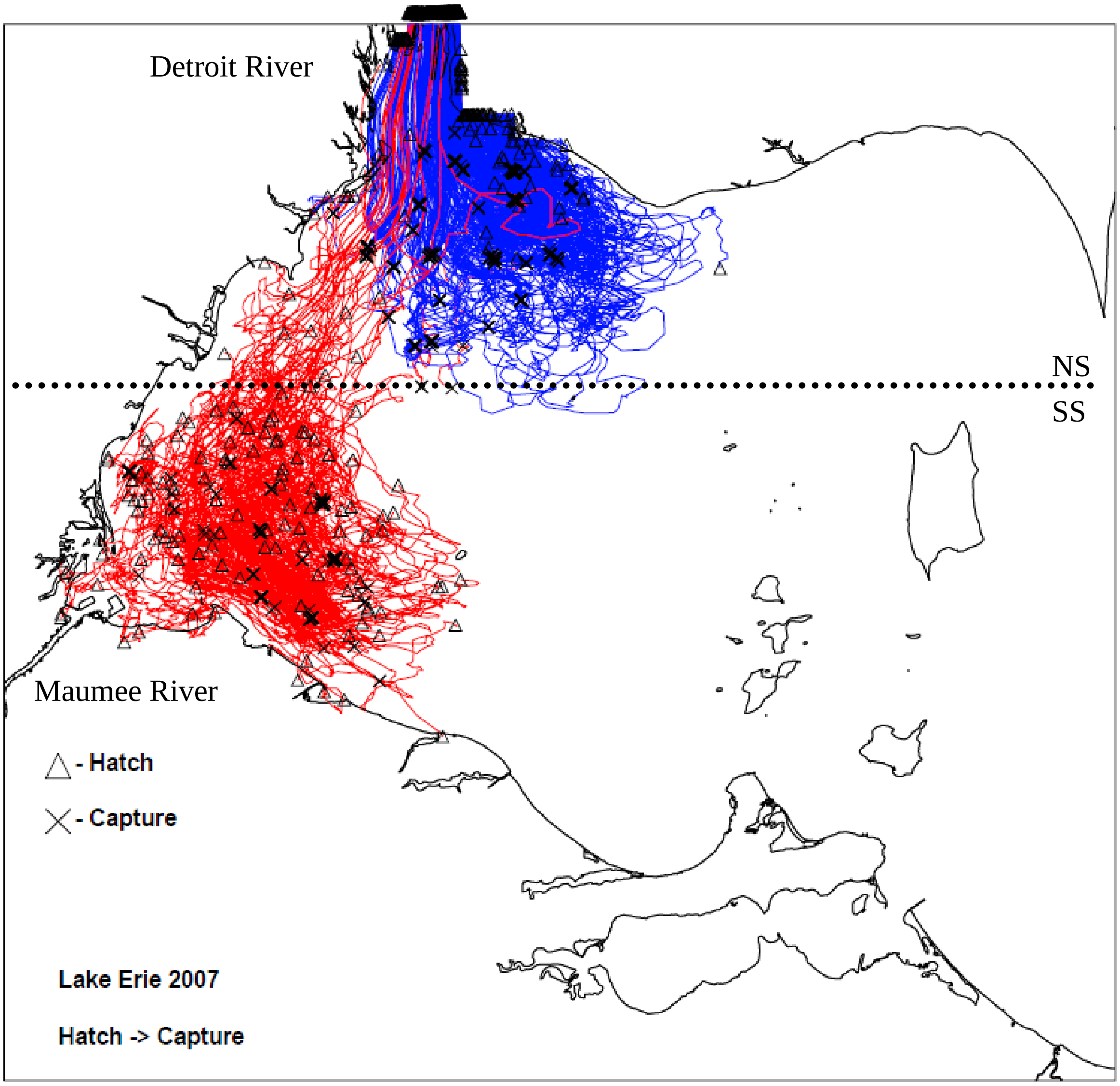
Detroit River
NS
SS
Maumee River
- Hatch
- Capture
Lake Erie 2007
Hatch -> Capture
