# Supplementary material for: Particle Backtracking Improves Breeding Subpopulation Discrimination and Natal-Source Identification in Mixed Populations
Source: PLoS One. 2015 Mar 23;10(3):e0120752. doi: 10.1371/journal.pone.0120752 (PMC4370746; doi:10.1371/journal.pone.0120752)
Supplement: S2 Appendix — (PDF) [file pone.0120752.s002.pdf]

S2 Appendix Genetic diversity indices based on larvae less than 8 mm TL and including only the seven loci in HWE

Number of genotypes (N) and alleles (N_A) and observed (H_O) and expected (H_E) heterozygosity for the seven microsatellite loci (Li et al. 2007) used to genotype larval yellow perch (YP) collected in the western basin of Lake Erie in 2006 ($N_{NS\ larvae}$=203, $N_{SS\ larvae}$=39) and 2007 ($N_{NS\ larvae}$=320, $N_{SS\ larvae}$=44). Groups are denoted by assigned hatch site followed by the year of collection.

		Locus						
**Groups**		**YP85**	**YP41**	**YP109**	**YP55**	**YP110**	**YP81**	**YP99**
NS06	N	158	167	131	147	144	160	**149**
	N_A	18	7	24	9	8	9	**14**
	H_O	0.77	0.63	0.81	0.51	0.11	0.67	**0.82**
	H_E	0.84	0.56	0.93	0.46	0.10	0.56	**0.85**
SS06	N	29	**33**	26	**33**	34	**27**	27
	N_A	10	**6**	18	**5**	3	**11**	10
	H_O	0.55	**0.88**	0.85	**0.79**	0.12	**0.44**	0.78
	H_E	0.74	**0.66**	0.94	**0.64**	0.17	**0.75**	0.86
NS07	N	**292**	300	**295**	311	290	303	298
	N_A	**22**	7	**30**	7	10	6	16
	H_O	**0.76**	0.64	**0.82**	0.61	0.12	0.61	0.88
	H_E	**0.79**	0.60	**0.94**	0.53	0.13	0.59	0.86
SS07	N	30	41	33	40	38	37	34
	N_A	16	5	21	5	5	5	11
	H_O	0.93	0.61	1.00	0.73	0.08	0.65	0.91
	H_E	0.89	0.57	0.95	0.59	0.13	0.54	0.85

Note: Data in bold denotes deviations from HWE (following Bonferroni correction).
