# Supplementary material for: Particle Backtracking Improves Breeding Subpopulation Discrimination and Natal-Source Identification in Mixed Populations
Source: PLoS One. 2015 Mar 23;10(3):e0120752. doi: 10.1371/journal.pone.0120752 (PMC4370746; doi:10.1371/journal.pone.0120752)
Supplement: S3 Appendix — (PDF) [file pone.0120752.s003.pdf]

S3 Appendix DAPC results

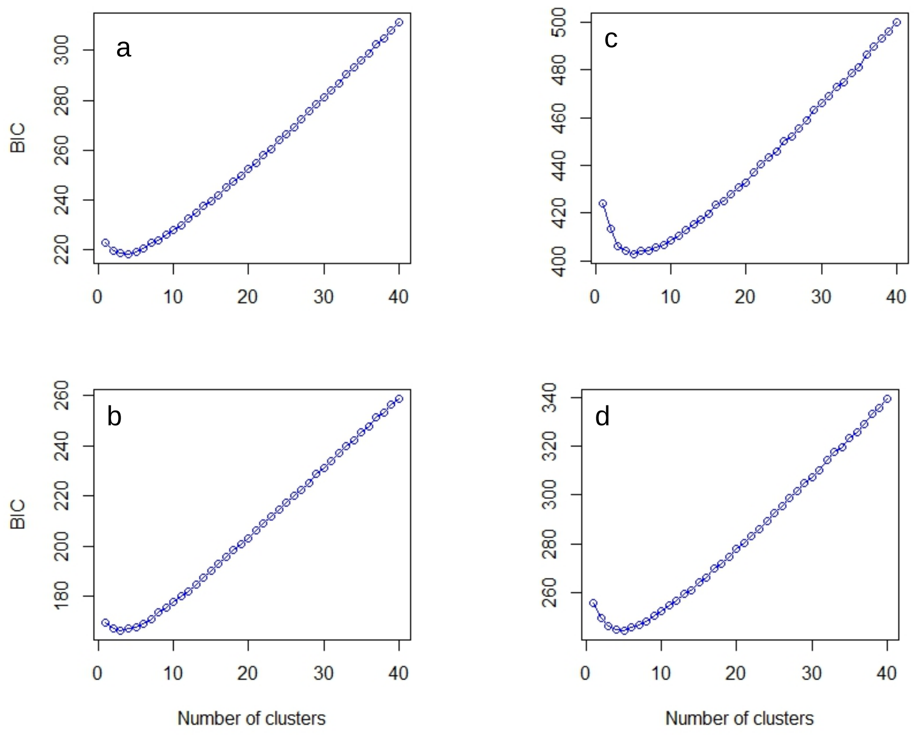

Results of k-means clustering for a) all 2006 larvae, b) 2006 larvae less than 8 mm TL and with a >90% hatch location certainty, c) all 2007 larvae, and d) 2007 larvae less than 8 mm TL and with a >90% hatch location certainty. K-means clustering searches for the clusters of each size that minimize the Bayesian Information Criterion (BIC).

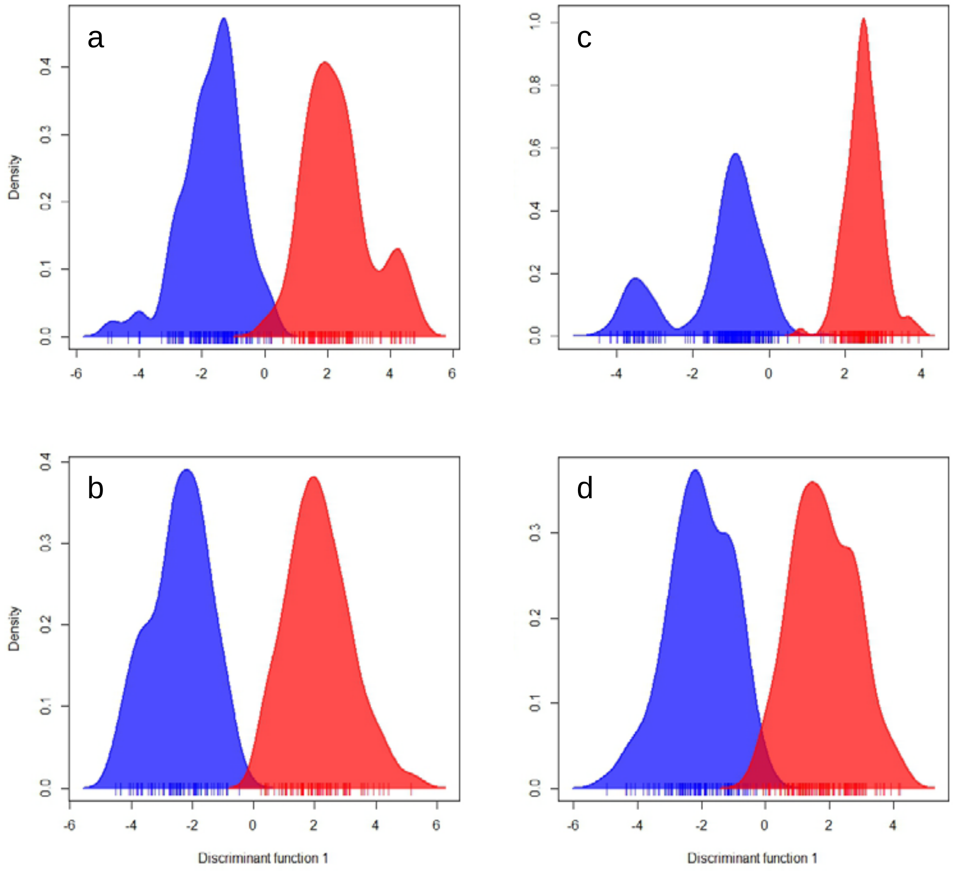

Density plot of individuals against value of first discriminant function for a) all 2006 larvae, b) 2006 larvae less than 8 mm TL and with a >90% hatch location certainty, c) all 2007 larvae, and d) 2007 larvae less than 8 mm TL and with a >90% hatch location certainty
