# Supplementary material for: Particle Backtracking Improves Breeding Subpopulation Discrimination and Natal-Source Identification in Mixed Populations
Source: PLoS One. 2015 Mar 23;10(3):e0120752. doi: 10.1371/journal.pone.0120752 (PMC4370746; doi:10.1371/journal.pone.0120752)
Supplement: S4 Appendix — (PDF) [file pone.0120752.s004.pdf]

S4 Appendix Larval assignment and juvenile classification based on 12 microsatellite loci

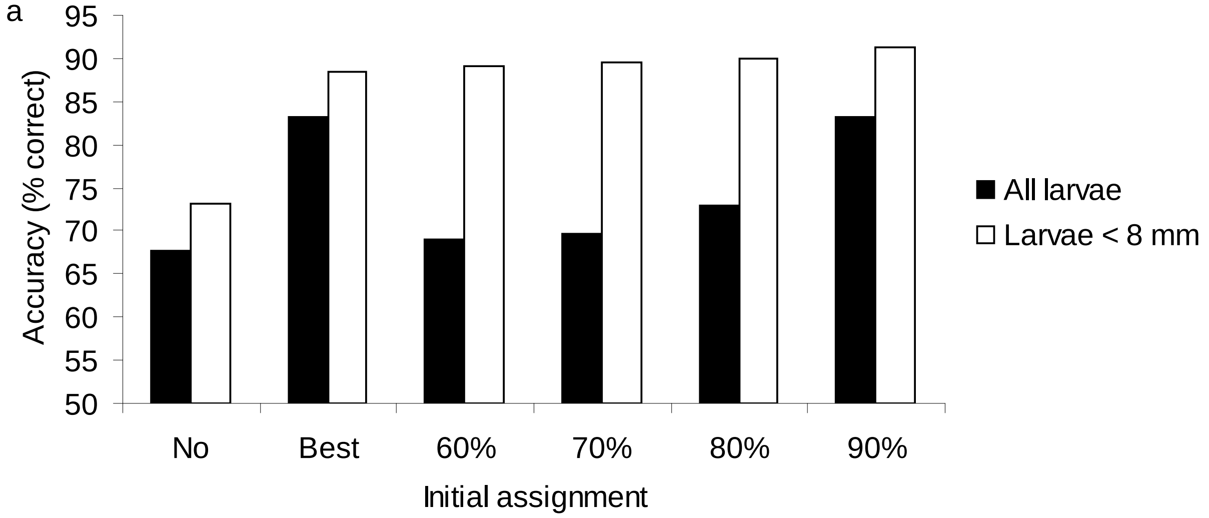

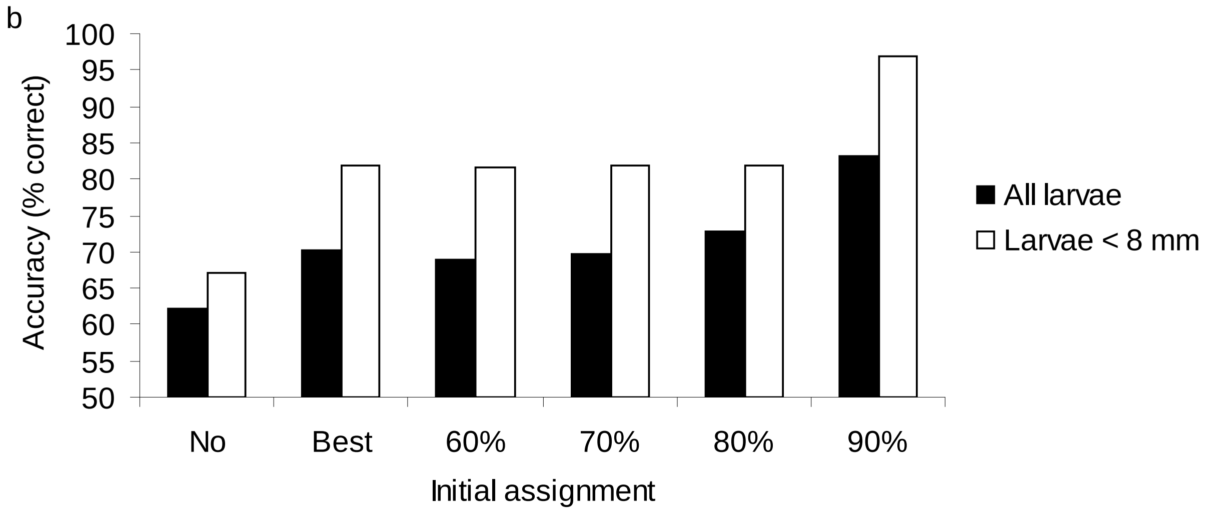

Larval yellow perch self-assignment accuracy in western Lake Erie based on 12 microsatellite loci a) during 2006 and b) during 2007. For initial assignment: No: null assignments based on capture location;, Best = best assignment after backtracking revision, 60, 70, 80, 90% = assignment after backtracking revision with 60, 70, 80, and 90% levels of certainty in source origin

Juvenile yellow perch assignment results in western Lake Erie using 12 microsatellites for 2006 (top) and 2007 (bottom). Rows indicate certainty of larval initial assignment: No: null assignments based on capture location; Best = best assignment after backtracking revision; 60, 70, 80, 90% = assignment after backtracking revision with 60, 70, 80, and 90% levels of certainty in source origin. Larvae of all sizes were included unless marked as < 8 mm

	% Excluded	% Failed	% NS	% SS
No	0	38	39	24
Best	0	14	67	18
No < 8 mm	0	27	43	29
Best < 8 mm	0	14	70	16
60% < 8 mm	0	15	71	9
70% < 8 mm	0	11	74	15
80% < 8 mm	0	13	71	16
90% < 8 mm	0	11	74	15
No	3	48	34	14
Best	2	34	47	18
No < 8 mm	4	35	34	28
Best < 8 mm	4	23	56	17
60% < 8 mm	4	23	56	11
70% < 8 mm	3	23	56	17
80% < 8 mm	3	22	51	23
90% < 8 mm	7	4	86	4

A total of n = 119 juveniles and n = 167 juveniles were analyzed during 2006 and 2007. Juveniles with < 30 % likelihood of originating from wither population were “excluded;” juveniles with a probability of assignment between 30 % and 70 % were considered “failed;” and juveniles with a probability > 70 % were assigned to “NS” or “SS”
